# Antioxidant, Anti-Cholinesterase, and Neuroprotective Properties of *Morus alba* and *Morus nigra* Extracts

**DOI:** 10.3390/antiox15040510

**Published:** 2026-04-20

**Authors:** Emanuela Nani Pohrib, Andreia Corciova, Oana Cioanca, Lucian Hritcu, Monica Hancianu, Andreea-Maria Mitran, Ana Flavia Burlec, Alexandra-Mara Cimpanu, Crina-Maria Isac, Riana Huzum, Ecaterina Danu, Cornelia Mircea

**Affiliations:** 1 Faculty of Pharmacy, Grigore T. Popa University of Medicine and Pharmacy Iasi, 16 University Street, 700115 Iasi, Romania; emanuela_t_pohrib@d.umfiasi.ro (E.N.P.); oana.cioanca@umfiasi.ro (O.C.); andreea-maria.mitran@umfiasi.ro (A.-M.M.); ana-flavia.l.burlec@umfiasi.ro (A.F.B.); cornelia.mircea@umfiasi.ro (C.M.); 2 Faculty of Biology, Alexandru Ioan Cuza University, B-dul Carol I, No. 11, 700506 Iasi, Romania; hritcu@uaic.ro (L.H.); alexandra.cimpanu@student.uaic.ro (A.-M.C.); crina.isac@student.uaic.ro (C.-M.I.); 3 Faculty of Medicine, Grigore T. Popa University of Medicine and Pharmacy Iasi, 16 University Street, 700115 Iasi, Romania; riana-maria.huzum@umfiasi.ro (R.H.); mg-rom-30837@students.umfiasi.ro (E.D.)

**Keywords:** *Morus alba*, *Morus nigra*, cholinesterase, neuroprotection

## Abstract

The *Morus* genus comprises several tree species whose fruits are used in human nutrition, while the leaves and roots are used in traditional medicine. The aim of this study was to highlight the antioxidant, cholinesterase inhibitory, and neuroprotective effects of hydroalcoholic extracts from *Morus alba* (MAE) and *Morus nigra* (MNE) leaves. RP-UHPLC-PDA analysis of extracts revealed the presence of polyphenols in higher quantities in MNE extract compared to MAE. Both extracts demonstrated antioxidant properties in the hydroxyl radical scavenging and lipid peroxidation inhibition assays. MNE exhibited a superior antioxidant capacity compared to MAE; the IC_50_ values for the inhibition of plasma lipid oxidation assay were 25.31 ± 2.54 µg/mL for MNE and 29.85 ± 0.97 µg/mL for MAE. Both extracts showed cholinesterase inhibitory activity. The IC_50_ values for acetylcholinesterase inhibition were 24.34 ± 0.86 µg/mL for MNE and 46.87 ± 2.16 µg/mL for MAE. The inhibitory potency of MNE was comparable to that of galantamine, which was used as standard. Both extracts reversed, in a dose-dependent manner, the scopolamine-induced cognitive impairment and behavioural alterations in scopolamine-treated zebrafish (*Danio rerio*) as evaluated by the Y-maze test, novel tank diving test, and novel object recognition test.

## 1. Introduction

*Morus alba* (white mulberry), *Morus nigra* (black mulberry) and *Morus rubra* (red mulberry) are the most important species within the *Morus* genus, belonging to the *Moraceae* family. Leaves, fruits, roots and stems from these species are used in traditional medicine, and fruits are used as food. Mulberry leaves are used in sericulture farms or to feed animals [1]. Water extracts or dried alcoholic extracts from leaves have been used in traditional medicine to treat diabetes [2], high blood pressure [3], hyperlipidaemia [4], inflammatory diseases, infections [5], and liver diseases [4]. Many studies indicate the antioxidant effects of leaf extracts due to their high content in phenolic acids, flavonoids and alkaloids [6].

Morin is a polyphenol, originally isolated from *Morus* species, but which can also be found in other plants. It is extracted from leaves, fruits and stems and presents antioxidant, antibacterial, antidiabetic, anti-inflammatory, antitumoral, and neuroprotective effects. Morin contains five hydroxyl groups able to release hydrogen and to stabilise hydroxyl radicals [7].

Morin reduces oxidative stress, neuroinflammation, and mitochondrial dysfunction, and improves neurotransmitter levels. The neuroprotective effects of morin are explained by its decreasing of oxidative stress, inflammation, excitotoxicity, calcium dysregulation, neurotransmitter alterations, protein modifications, and enzymatic inhibition [7,8].

Reactive oxygen species (ROS) are generated in living organisms as a result of cellular respiration (in small quantities) and uncontrolled oxidative processes. Another major source of ROS is represented by primary immune defence mechanisms. Neutrophils, monocytes, and macrophages produce reactive oxygen and nitrogen species to destroy pathogenic microorganisms. However, excessive production of oxidants can lead to the degradation of lipids, proteins, nucleic acids, and carbohydrates, resulting in cellular damage, tissue injury, accelerated aging, and the development of pathological conditions such as cancer, atherosclerosis, Alzheimer’s disease, Parkinson’s disease, diabetes mellitus, and cardiovascular disorders [9].

Oxidative stress is a key factor in the development and progression of Alzheimer’s disease and is involved in neurodegenerative processes [10]. ROS include both free radicals (such as the superoxide anion radical O_2_•^−^, hydroxyl radical OH•, and various peroxyl and hydroperoxyl radicals) and non-radical species, including hydrogen peroxide (H_2_O_2_), ozone (O_3_), and peroxynitrite (ONOO^−^). Oxidative stress contributes to beta-amyloid plaque formation, abnormal tau protein phosphorylation, and neuronal dysfunction, thereby exacerbating Alzheimer’s disease progression [11]. Studies have shown that under hypoxic conditions, hydrogen peroxide (H_2_O_2_) and peroxynitrite (ONOO^−^) levels are significantly increased, accelerating the neurodegenerative process. Excessive ROS may induce mutations in mitochondrial DNA, lipid peroxidation, and opening of mitochondrial membrane channels, ultimately leading to mitochondrial dysfunction [12].

Cerebral endothelial dysfunction leads to brain hypoxia, oxidative stress, microglial activation with increased synthesis of proinflammatory cytokines, and neuroinflammation [12].

Alzheimer’s disease is a chronic neurodegenerative disorder characterized by a decline in cognitive function, memory loss, speech impairment, and behavioural problems. The prevalence of Alzheimer’s disease is continuously increasing, and in the last years a downward trend in the age of onset is observed. Consequently, there is a critical need to identify novel therapeutic options to mitigate the epidemiological impact and to alleviate the socio-economic burden associated with long-term patient care [13].

Alzheimer’s disease is determined by risk factors such as genetics, age, environmental factors, brain trauma, infections, vascular diseases and metabolic disorders. The mechanisms involved in the onset and worsening of Alzheimer’s disease are different and consist of the following: amyloid beta aggregation, hyperphosphorylation/abnormal aggregation of tau proteins, damage to the cholinergic system, accumulation of toxic metals in neurons, oxidative stress, neuroinflammation, mitochondrial dysfunction and neuron loss [14].

The cholinergic system involves acetylcholine, cholinergic receptors and enzymes that hydrolyse the neuromediator, such as acetylcholinesterase and butyrylcholinesterase. The involvement of the cholinergic system is suggested as the earliest of events in Alzheimer’s disease pathophysiology. The discovery of the cholinergic hypothesis as a mechanism involved in the pathology of Alzheimer’s disease has stimulated research into the identification of compounds that can reduce the activity of these enzymes. The “cholinergic hypothesis” suggests that degeneration of cholinergic neurons in the basal forebrain and impaired cholinergic neurotransmission in the cortex and hippocampus represent the primary causes of memory and cognitive deficits in patients with Alzheimer’s disease [14,15].

Increased acetylcholinesterase activity leads to a reduction in acetylcholine levels in the brain. Acetylcholinesterase also exhibits non-cholinergic actions that contribute to disease progression, including stimulation of beta-amyloid aggregation, formation of neurofibrillary aggregates, and development of senile plaques [16].

The deficiency of acetylcholine in the brains of patients with Alzheimer’s disease is also determined by the reduction in choline transferase activity resulting in decreased synthesis of this neurotransmitter.

Alzheimer’s disease therapy is complex and is based on the use of drugs from different therapeutic classes that act by inhibiting cholinesterases, reducing the synthesis and deposition of beta-amyloid, blocking NMDA receptors, and, as a general mechanism, reducing oxidative stress [17].

The drugs currently used to treat cognitive deficit and Alzheimer’s disease are quite few, and the therapeutic benefits are relatively moderate considering patients’ expectations. Moreover, the aging population and the increasing prevalence of Alzheimer’s disease have intensified research in this field to identify compounds with good pharmacological effects and minimal side effects.

Cholinesterase inhibitors are among the drugs approved by the FDA for the treatment of Alzheimer’s disease and are considered the most effective therapy currently available. These drugs improve symptoms related to language, thinking, attention, memory, and judgment by inhibiting the hydrolysis of acetylcholine by acetylcholinesterase, resulting in increased acetylcholine concentrations in the synaptic cleft and enhanced cholinergic neurotransmission. Recent studies suggest that the long-term use of these medications may also influence disease progression by increasing neurotransmitter availability, reducing inflammation, and delaying cerebral astrogliosis [14,18].

Cholinesterase inhibitors currently used in therapy increase the concentration of acetylcholine in the synaptic cleft and improve symptoms. In contrast, plant extracts may have a dual effect by inhibiting cholinesterases and reducing oxidative stress, thus influencing two of the mechanisms involved in the onset of Alzheimer’s disease. Many compounds that block or reduce the activity of cholinesterases are isolated from medicinal plants and usually are alkaloids, terpenoids, polyphenols, and phenolic acids [19].

To investigate the neuroprotective potential of the tested extracts in a relevant in vivo system, we employed a scopolamine (Sco)-induced cognitive impairment model in zebrafish (*Danio rerio*). Sco, a non-selective muscarinic acetylcholine receptor antagonist, is widely used to induce memory deficits and cholinergic dysfunction resembling key features of AD [20,21]. The zebrafish model is increasingly utilized in neuroscience research due to its well-conserved neurotransmitter systems, including the cholinergic pathway, and its suitability for behavioural paradigms assessing learning, memory, and anxiety-like responses [22,23]. Moreover, the Sco-treated zebrafish paradigm has been extensively validated as a reliable experimental model for screening neuroprotective and procognitive agents, including natural compounds with antioxidant and anticholinesterase properties [24,25]. Therefore, this model represents a relevant and translational tool for evaluating the neuroprotective effects of *Morus* sp. extracts in the context of oxidative stress and cholinergic dysfunction.

Analysing data from previous studies on the involvement of oxidative stress and cholinergic mechanisms in neurodegeneration, the aim of the study is to evaluate the antioxidant (hydroxyl radical scavenging assay; lipid peroxidation inhibition assay), anticholinesterase and neuroprotective properties of two extracts obtained from the leaves of *Morus* sp. For the cholinesterase inhibition assay, both enzymes, acetylcholinesterase and butyrylcholinesterase, were tested. The neuroprotective effects of extracts were evaluated by applying behavioural tests on zebrafish (*Danio rerio*): Y-Maze Test, novel tank diving test (NTT), and novel object recognition test (NOR).

## 2. Materials and Methods

*Extracts preparation: Morus alba* and *Morus nigra* leaves were collected from their natural habitat in Iasi, Romania. Voucher specimens are deposited at the Department of Pharmacognosy, Faculty of Pharmacy, “Grigore T. Popa” University of Medicine and Pharmacy from Iasi, Romania. A total of 5 g of dried and powdered leaves were extracted with 100 mL ethanol–water 70:30 on water bath, at reflux, for 60 min. The extracts were dried using a rotary evaporator. Dried extracts (*Morus alba*—MAE, *Morus nigra*—MNE) were dissolved in dimethyl sulfoxide (DMSO) in order to obtain solutions with concentrations ranging from 0.078125 to 10 mg/mL, which were subsequently subjected to analysis.

Morin and the standards were tested at the same concentration as the plant extracts.

### 2.1. Chemical Analysis

*RP-UHPLC-PDA analysis*: The dried extract was dissolved in methanol, and 15 µL of solution was injected. Mobile phase: A gradient system consisting of A (acetonitrile) and B (0.1% acetic acid in ultrapure distilled water) was employed, starting at 10% A, increased to 15% A over 8 min, then to 30% A over the following 7 min, followed by an increase to 80% over 5 min, and finally returned to the initial conditions for the last 5 min. The flow was 0.5 mL/min in the first 8 min, then 0.8 mL/min until the end of run. Simultaneous detection at 254 nm, 280 nm, 330 nm and 521 nm was used for flavonoids and polyphenols. The obtained chromatogram was integrated in Chromeleon 7.2 software and compared to the spectral library that included standards of pure quality and acquired from Sigma-Aldrich, Steinheim, Germany. The liquid chromatography coupled with photodiode array detection used a Thermo Ultimate 3000 system that included an autosampler and a Kinetex C18 column (100 A, 150 × 4.6 mm), manufactured by Phenomenex (Torrance, CA, USA).

The identification took into consideration the retention time, match factor (of at least 900 of 1000) and UV spectra for the following compounds: caffeic acid, gallic acid, chlorogenic acid, trans-ferulic acid, cinnamic acid, rutoside, quercetin, isoquercetin (quercetin-3-beta-D-glucoside), hyperoside, apigenin, luteolin, gallocatechin, (epi)-catechin, quercetin-3-arabinoside, cyanidin-3-glucoside, kaempferol, luteolin-7-glucoside, morin.

### 2.2. Antioxidant Tests

*Hydroxyl Radical Scavenging Assay*: A volume of 0.225 mL of the sample solution was mixed with 0.750 mL of iron (II) sulphate solution (1.5 mM), 0.9 mL of sodium salicylate solution (20 mM), and 0.525 mL of hydrogen peroxide solution (6 mM). After 30 min, the absorbance of the sample was read at 562 nm compared to the sample blank [26]. The positive control solution was analysed under the same experimental conditions, with ascorbic acid used as standard.

The hydroxyl radical scavenging activity was calculated according to the formula

Scavenging activity % = 100 × (Ac − As)/(Ac)

where Ac represents the absorbance of the control solution, and As represents the absorbance of the sample or standard solution.

*Lipid peroxidation inhibition assay*: A volume of 0.094 mL serum was mixed with 0.05 mL sample, 0.006 mL of 2 mM solution CuCl_2_ and was incubated for 24 h at 37 °C. After 24 h, the next solutions were added: 0.2 mL of 8.1% sodium dodecyl sulphate solution, 1.5 mL of acetate buffer 3.5 M (pH 4) and 1.5 mL of 0.8% thiobarbituric acid solution. The mixture was boiled for 1 h, and was cooled and centrifugated at 2000 RPM at 4 °C for 10 min. The sample, control and standard supernatant are separated, and the absorbance was read against blank at 532 nm [27]. Ascorbic acid was used as standard compound.

The capacity of the tested compounds or standard to block the lipid peroxidation was calculated according to the formula

Inhibition % = 100 × (Ac − As)/(Ac)

where Ac represents the absorbance of the control solution, and As represents the absorbance of the sample or standard solution.

### 2.3. Cholinesterase Inhibition Tests

*Cholinesterase inhibition assay* (modified Ellman’s method): A volume of 0.56 mL of 0.1 M phosphate buffer (pH 8) was mixed with 0.08 mL of 0.1 M DTNB solution, 0.08 mL sample (or standard), and 0.08 mL of 1 UI/mL enzyme solution (acetylcholinesterase or butyrylcholinesterase). The mixture was kept for 10 min at 25 °C, and after that 0.04 mL acetylthiocholine iodide solution, or 0.04 mL butyrylthiocholine iodide solution, were added. The absorbance was read at 412 nm for 3 min [28]. Galantamine was used as standard.

The enzyme inhibition was calculated according to the formula

Inhibition% = 100 × (Ac − As)/(Ac)

where Ac represents the absorbance of the control solution, and As represents the absorbance of the sample or standard solution.

Each experiment was performed in triplicate, and the mean and standard deviation were calculated.

### 2.4. Behavioural Studies

*Animals*: At the beginning of the study, 72 adult zebrafish (*Danio rerio*), wild-type short-fin strain of both sexes (ratio 50:50 male:female, 3–4-month-old, and 3–4 cm-long), were acclimatized in the experimental room for at least 14 days. Fish were kept on a 14/10 h light/dark cycle in 24 L thermostated (26 ± 1 °C) tanks, under water filtration and aeration (7.20 mg O_2_/L) using TetraTec^®^ air pumps (Tetra, Melle, Germany). Animals were fed twice a day with Norwin Norvitall flake (Norwin, Gadstrup, Denmark). Acclimatized zebrafish (10 fish/group) were randomly assigned to the next groups: control, scopolamine (Sco, 100 μM), morin (20 μM), MAE (exposed to *Morus alba* extract—1 and 10 μg/L), MNE (exposed to *Morus nigra*—1 and 10 μg/L), and reference compounds. The doses for extracts and different tested compounds were selected according to our previous studies. The control group was immersed only in unchlorinated water with a 1% DMSO solution. Galantamine (GAL, 3.5 μM) was used as a reference compound. The animals were purchased from the European Zebrafish Resource Center at the Institute of Toxicology and Genetics, Germany.

The animal study protocol was approved by the Ethics Committee on Animal Research of the Alexandru Ioan Cuza University of Iasi, Faculty of Biology (Iasi, Romania) under license no. 12/8 January 2026, and it fully complied with Directive 2010/63/EU of the European Parliament and of the Council of 22 September 2010 on the safety of animals. The health status and the well-being of all animals involved in the research were tested regularly during behavioural tests. No procedures caused serious pain or long-lasting damage to the zebrafish, and no experimental subject died during the experimental procedures.

The 8-day treatment period was selected based on previously reported protocols using scopolamine-induced cognitive impairment in zebrafish, where subchronic exposure (typically 7–10 days) is necessary to ensure sufficient accumulation and pharmacodynamic action of test compounds, as well as to obtain stable and reproducible behavioural responses [20,29]. Such experimental designs are widely employed to evaluate the neuroprotective and procognitive effects of plant-derived compounds in vivo, allowing the assessment of cumulative effects on memory, anxiety-like behaviour, and cholinergic function.

*Y-Maze Test*: The Y-maze test consisted of two trials separated by a 1 h interval. During the first trial, 1 h after treatment with tested extracts, the fish could freely swim in the start arm and the other arm for 5 min. The third arm (novel arm) was blocked by a dividing wall. In the second trial, the new arm is opened, and the fish could explore all three arms for 5 min, including the novel environment constituted by the novel arm. Fish were placed in different arms as starting points, and the maze was rotated in each experiment to randomize the maze cues. The apparatus consisted of a Y-maze glass tank with three identical arms (25 cm long, 8 cm wide, and 15 cm high) arranged at 120° angles, filled with 3 L of the home aquarium water. The water in the Y-maze was 13 cm. Explicit geometric shapes (squares, circles, and triangles) were placed on the outer walls and visible from the inside. The water was changed between groups and trials. The MAE and MNE (1 and 10 µg/L) and morin (20 µM) were diluted with 1% DMSO solution and administered to zebrafish by immersion for 1 h once daily for 8 days, while Sco (100 µM) was administered 30 min before each behavioural test. The control group was immersed only in unchlorinated water with a 1% DMSO solution. Galantamine (GAL, 3.5 μM) was used as a reference compound. Zebrafish behaviour was recorded using a Logitech C922 Pro HD Stream webcam (Logitech, Lausanne, Switzerland). The behaviour was fully analysed using ANY-Maze^®^ software v. 7.49 (Stoelting CO, Wood Dale, IL, USA), assessing time spent in the novel arm (% of total arm time), total distance travelled (m), and turn angle [30,31].

*Novel tank diving test (NTT)*: The fish were individually placed in the testing tank, and their behaviour was recorded for 6 min using a webcam positioned 40 cm in front of the tank. The groups of animals are the same as in the Y-maze test. The testing apparatus consisted of a trapezoidal glass tank filled with 1.5 L of home tank water and having the following dimensions: 23.9 cm along the bottom × 28.9 cm at the top × 15.1 cm high with 15.9 cm along the diagonal side, 7.4 cm wide at the top, and 6.1 cm wide at the bottom. The tank was virtually divided into the top zone and the bottom zone. To measure anxiety-like behaviour and the locomotor activity of the zebrafish, we used the behavioural endpoints described previously by Cachat et al. [32,33]. The zebrafish swimming behaviour during the in vivo tasks was recorded with a Logitech C922 Pro HD Stream webcam (Logitech, Lausanne, Switzerland), and the recordings were analysed using ANY-maze software v6.3 (Stoelting Co., Wood Dale, IL, USA).

*Novel object recognition test (NOR)*: The experimental apparatus consists of a 20 L glass tank (30 × 30 × 30 cm) filled with 6 cm of water. Before training, each animal was habituated to the apparatus in the absence of the objects for 5 min twice a day (5 h interval between habituation sessions) over 3 consecutive days. On the 4th day, in the training phase (with 1 h retention interval), the animals were exposed to two identical red cubes for 10 min. In the test phase, a novel object (N, green cube) replaced one of the copies of the familiar objects (F, red cube), and the exploration time of each object was evaluated for 10 min. The preference percentages were the behavioural parameters evaluated in this test. The preference percentages were calculated as [time of exploration of NO/time of exploration of FO + time of exploration of NO × 100] [34]. The zebrafish swimming behaviour during the in vivo tasks was recorded with a Logitech C922 Pro HD Stream webcam (Logitech, Lausanne, Switzerland), and the recordings were analysed using ANY-maze software v7.49 (Stoelting Co., Wood Dale, IL, USA).

**Reagents**: Morin, hydrogen peroxide, sodium salicylate, copper chloride, sodium dodecylsulphate, thiobarbituric acid, sodium diphosphate, sodium monophosphate, 5,5′-bis-2-nitrobenzoic acid (DTNB), acetylcholinesterase from *Electrophorus electricus* (electric eel), type V-S, butyrylcholinesterase from horse serum, acetylthiocholine iodide, butyrylthiocholine iodide, bovine serum, galantamine, imipramine, and scopolamine were purchased from Sigma-Aldrich (Steinheim, Germany). Ethyl alcohol, dimethyl sulfoxide, acetonitrile, iron sulphate (II), acetic acid, and methanol were purchased from Merck, Darmstadt, Germany. Ultrapure water was obtained from the SG Water Ultra Clear TWF water purification system (Barsbüttel, Germany).

### 2.5. Statistical Analysis

The results are expresses as mean ± standard deviation. One-way analysis of variance (ANOVA) was performed to evaluate the statistically significant differences between samples, and *p*-value below 0.05 was considered as statistically significant. The statistical analysis was computed using SPSS software v27.

## 3. Results

### 3.1. Chemical Analysis

**Figure 1:**
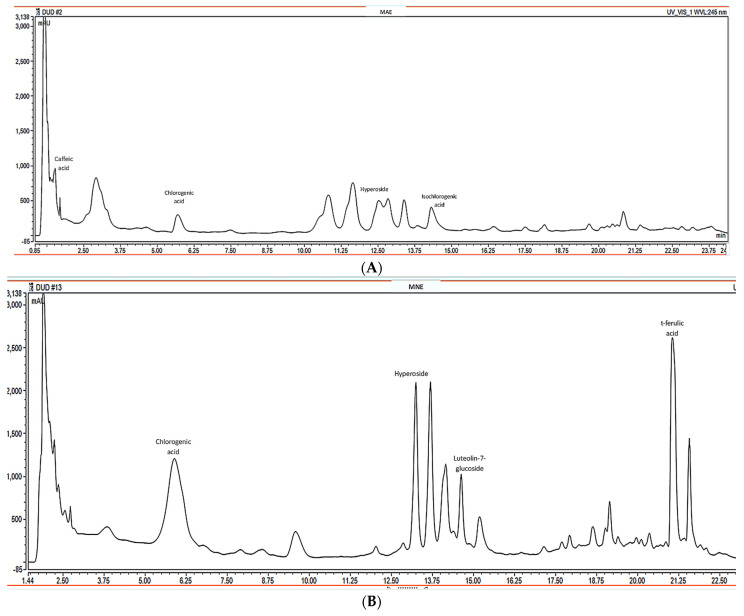
The chromatograms from RP-UHPLC-PDA analysis of extracts: (**A**)—*Morus alba* extract (MAE); (**B**)—*Morus nigra* extract (MNE).

The RP-UHPLC-PDA analysis used to characterize the hydroalcoholic MAE and MNE extracts revealed the presence of polyphenols, with the most important constituents summarized in Table 1 and Figure 1.

### 3.2. Antioxidant Evaluation

#### 3.2.1. Hydroxyl Radical Scavenging Assay

*The hydroxyl radical* is a reactive oxygen species (ROS) generated in vivo, playing a critical role in the uncontrolled oxidation of lipids, proteins, carbohydrates and nucleic acids. In vivo, the hydroxyl radical is generated via the Fenton and Haber–Weiss reactions, catalysed by transition metal ions such as iron or copper. This species is recognized as one of the most aggressive free radicals [35].

**Figure 2:**
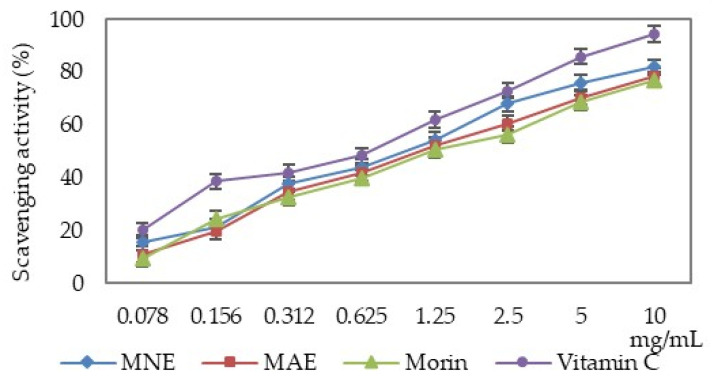
Determination of the hydroxyl radical scavenging activity (MAE—*Morus alba* extract, MNE—*Morus nigra* extract).

MAE, MNE and morin have the ability to neutralise the hydroxyl radicals depending on the solution concentration. Their scavenging properties are lower than that of ascorbic acid (Figure 2).

The hydroxyl radical scavenging capacity of the two extracts and morin was over 50%; thus, the IC_50_ values were calculated and are presented in Table 2.

When comparing the IC_50_ values, it can be seen that MNE exhibits a 15.67% lower scavenging activity compared to vitamin C, but 12.60% higher compared to MAE and 21.11% higher compared to morin. MAE is 26.29% less active compared to vitamin C.

#### 3.2.2. Lipid Peroxidation Inhibition Assay

**Figure 3:**
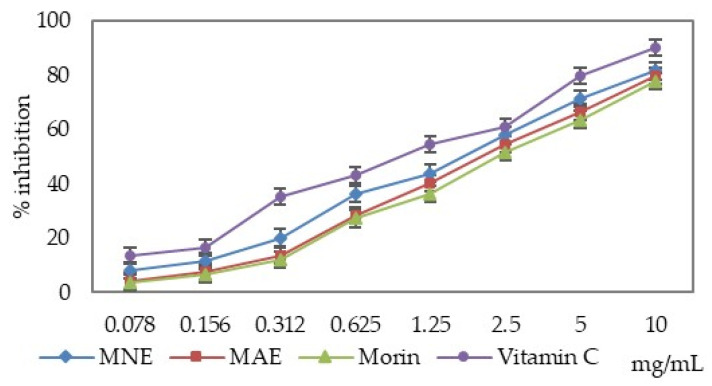
The results for lipid peroxidation inhibition assay (MAE—*Morus alba* extract, MNE—*Morus nigra* extract).

The analysed extracts and morin reduced lipid peroxidation induced by copper ions. The rate of inhibition depends on the sample concentration. Both extracts exhibited stronger inhibitory activity than morin, but lower activity than ascorbic acid (Figure 3, Table 2).

When comparing the IC_50_ values, it can be noticed that MNE exhibits a 15.20% higher activity compared to MAE and is 36.54% more active than morin (Table 2). MNE is 4.78% less active compared to vitamin C, while MAE and morin are less active compared to vitamin C by 19.26% and 30.26%, respectively.

### 3.3. Cholinesterase Inhibition Assay

Acetylcholinesterase (AChE; E.C. 3.1.1.7) is a membrane serine hydrolase that catalyses the hydrolysis of acetylcholine to choline and acetic acid [36]. In addition to its effect on the cholinergic system, acetylcholinesterase also plays a role in haematopoiesis and thrombopoiesis [37].

Butyrylcholinesterase (BChE; E.C. 3.1.1.8), also referred to as pseudocholinesterase, is a serine protease that catalyses the hydrolysis of acetylcholine and exhibits 54% structural similarity to acetylcholinesterase. This enzyme is primarily produced by glial cells [38].

**Figure 4:**
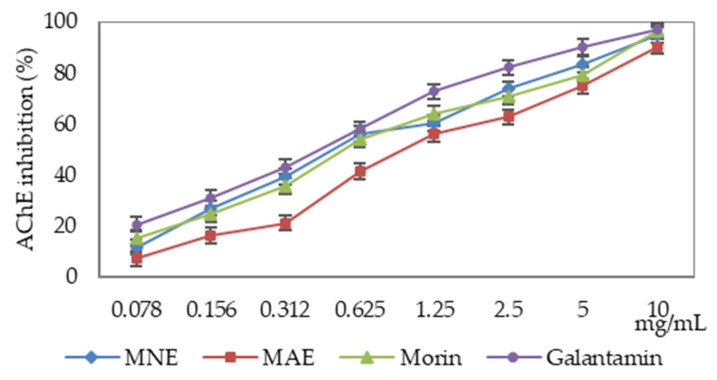
The results for acetylcholinesterase (AChE) inhibition assay (MAE—*Morus alba* extract, MNE—*Morus nigra* extract).

**Figure 5:**
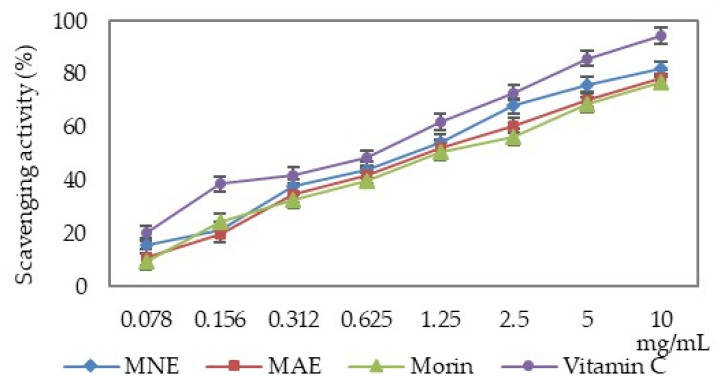
The results for butyrylcholinesterase (BChE) inhibition assay (MAE—*Morus alba* extract, MNE—*Morus nigra* extract).

*Morus* sp. extracts and morin reduce the cholinesterase activity depending on the used enzyme and the sample concentration (Figure 4 and Figure 5, Table 2). The sample’s activity was compared with galantamine, a drug used to treat Alzheimer’s disease.

The cholinesterases were differently influenced by the extracts and morin, with acetylcholinesterase activity being more markedly reduced than that of butyrylcholinesterase.

The extract from *Morus nigra* leaves is more active compared to morin, but does not exceed the inhibition capacity of galantamine. Comparison of IC_50_ values indicated that that MNE was 13.20% less active than galantamine, whereas MAE exhibited approximatively twofold lower activity. In comparison with morin, MNE exhibited 10.27% higher activity, whereas MAE demonstrated 74.62% lower activity (Table 2).

### 3.4. Behavioural Tests

The neuroprotective effects of *Morus* sp. extracts and morin have been evaluated by three behavioural tests on zebrafish in special experimental conditions.

- *Effects on response to novelty and locomotion in the Y-maze test*

**Figure 6:**
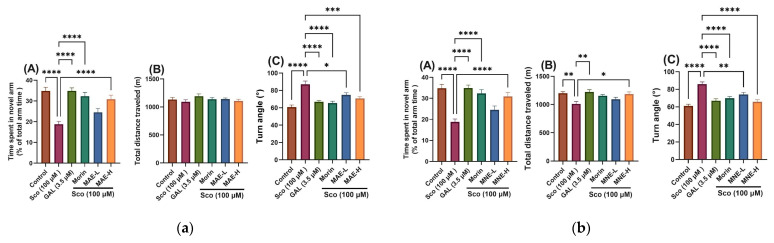
Assessment of response to novelty in Sco (100 µM) and samples-treated zebrafish in the Y-maze test: (**a**) MAE treatment at doses of MAE-L (1 μg/L) and MAE-H (10 μg/L); (**b**) MNE treatment at doses of MNE-L (1 μg/L) and MNE-H (10 μg/L). For each panel: (**A**) time spent in the novel arm (% of total arm time); (**B**) total distance travelled (m); (**C**) turn angle (°). Data are presented as means ± S.E.M. (n = 12 animals per group). * *p* < 0.01, ** *p* < 0.001, *** *p* < 0.0001, and **** *p* < 0.00001 (Tukey’s post hoc analyses). Galantamine (GAL, 3.5 μM) was used as a reference positive drug.

Spatial memory and the response to novelty in zebrafish was assessed using the Y-maze test [30]. The location in the Y-maze task was considered to be a memory index [31]. The results obtained in Y-maze testing are presented in Figure 6.

In order to evaluate the effects of MAE and MNE hydroalcoholic extracts on novelty-driven exploratory behaviour, zebrafish were assessed using the Y-maze test, and the percentage of time spent in the novel arm was analysed [30,31]. For MAE one-way ANOVA revealed a significant overall treatment effect on time spent in the novel arm [F(5, 66) = 14.03, *p* < 0.0001] (Figure 6a, panel A), total distance travelled [F(5, 66) = 1.12, *p* = 0.35] (Figure 6a, panel B), and turn angle [F(5, 66) = 13.51, *p* < 0.0001] (Figure 6a, panel C). In the same time for MNE one-way ANOVA revealed a significant overall treatment effect on time spent in the novel arm [F(5, 66) = 14.03, *p* < 0.0001] (Figure 6b, panel A), and turn angle [F(5, 66) = 14.87, *p* < 0.0001] (Figure 6b, panel C), whereas no significant effect was observed on total distance travelled [F(5, 66) = 4.959, *p* = 0.0006] (Figure 6b, panel B).

Post hoc analysis demonstrated that scopolamine (Sco, 100 μM) significantly reduced the percentage of time spent in the novel arm compared with the control group (*p* < 0.00001), confirming successful induction of cognitive impairment.

- *Effects of MAE, MNE and morin on anxiety in the NTT task*

**Figure 7:**
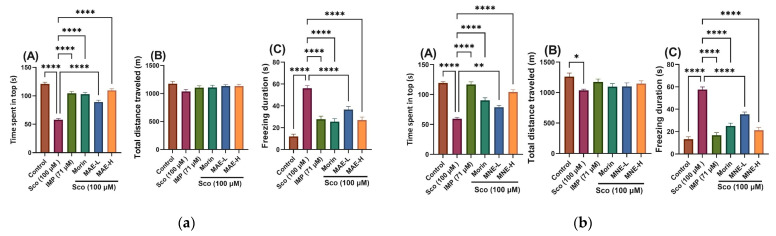
The effects of extracts and morin in scopolamine (Sco, 100 µM)-treated zebrafish on anxiety behaviour evaluated by the novel tank diving test (NTT): (**a**) MAE treatment at doses of MAE-L (1 μg/L) and MAE-H (10 μg/L); (**b**) MNE treatment at doses of MNE-L (1 μg/L) and MNE-H (10 μg/L). For each panel: (**A**) time spent in the top (s); (**B**) total distance travelled (m); (**C**) freezing duration (s). Data are means ± S.E.M. (n = 12). For Tukey’s post hoc analyses, * *p* < 0.01, ** *p* < 0.001, **** *p* < 0.00001. Imipramine (IMP, 71 µM) was used as a positive reference drug.

The NTT test evaluated the novelty-provoked anxiety by measuring the time spent in the top (s) and freezing duration (s), and the locomotion by total distance travelled (m) (Figure 7).

In the NTT, the zebrafish exhibited robust behavioural responses to novelty-provoked anxiety.

The novel tank test (NTT) was used to evaluate anxiety-like behaviour induced by novelty. One-way ANOVA revealed MAE treatment significantly modified the time spent in the top zone of the tank [F(5, 66) = 56.39, *p* < 0.0001] (Figure 7a, panel A) and freezing duration [F(5, 66) = 31.02, *p* < 0.0001] (Figure 6a, panel C); MNE had a significant effect of treatment on time spent in the top zone of the tank [F(5, 66) = 43.04, *p* < 0.0001] (Figure 7b, panel A) and freezing duration [F(5, 66) = 48.80, *p* < 0.0001] (Figure 6b, panel C). For MAE the total distance travelled [F(5, 66) = 1.776, *p* = 0.13] (Figure 7a, panel B) remained unchanged, and for MNE total distance travelled was not significantly affected [F(5, 66) = 2.460, *p* = 0.0419] (Figure 7b, panel B). Scopolamine (Sco, 100 μM) significantly reduced the time spent in the top zone compared with the control group (*p* < 0.00001), indicating increased anxiety-like behaviour. Additionally, Sco markedly increased freezing duration (*p* < 0.00001), demonstrating a hypolocomotor effect relative to controls. The effects of morin were similar to imipramine, which was used as positive control.

The absence of significant differences in the total distance travelled indicates that these behavioural alterations were not associated with major changes in general locomotor activity.

- *Effects of MAE, MNE and morin on recognition memory in the novel object recognition test (NOR)*

**Figure 8:**
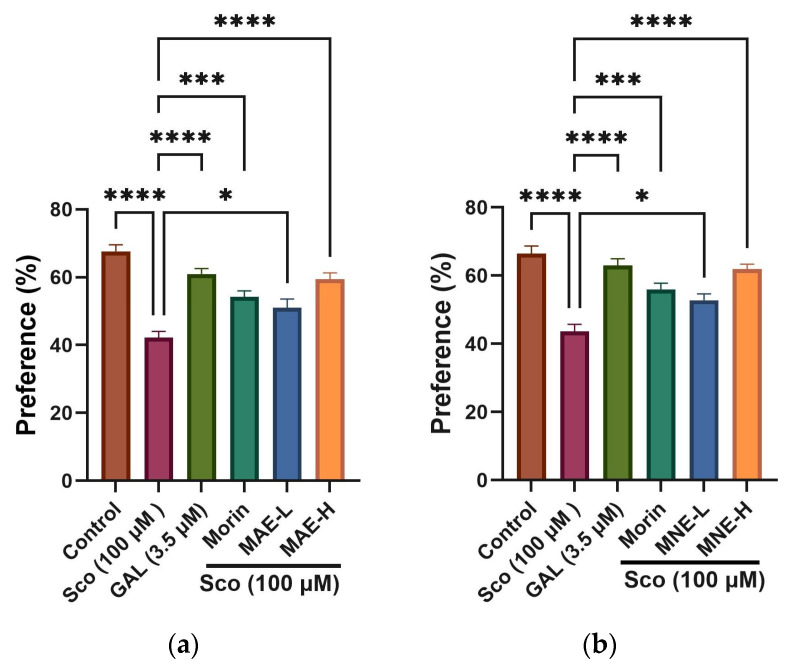
The effects of extracts and morin in scopolamine (Sco, 100 µM)-treated zebrafish on the preference percentage evaluated within the novel object recognition test (NOR): (**a**) MAE treatment at doses of MAE-L (1 μg/L) and MAE-H (10 μg/L); (**b**) MNE treatment at doses of MNE-L (1 μg/L) and MNE-H (10 μg/L). Data are means ± S.E.M. (n = 10). For Tukey’s post hoc analyses, * *p* < 0.01, *** *p* < 0.0001, and **** *p* < 0.00001. Galantamine (GAL, 3.5 µM) was used as a positive reference drug.

NOR is a commonly used behavioural assay for the investigation of memory performance in zebrafish. The results obtained in this test are presented in Figure 8.

In the novel object recognition (NOR) test, for both extracts, one-way ANOVA revealed a significant overall effect of treatment on preference percentages [F(5, 66) = 20.89, *p* < 0.0001] (Figure 8). Scopolamine (Sco, 100 μM) markedly reduced the preference for the novel object compared with the control group (*p* < 0.00001), indicating impaired recognition memory.

## 4. Discussion

Our previous studies indicate a different chemical composition for MAE and MNE extracts used in the present study. The quantity of polyphenols, expressed as mg gallic acid equivalents/g extract, is higher in MNE (89.46 ± 0.07) compared to MAE (62.53 ± 0.02). We noted the same difference for flavonoids, expressed as mg rutoside equivalents/g extract: 11.22 ±0.01 for MNE, and 7.16 ±0.01 for MAE [39].

The presence of compounds such as chlorogenic acid, caffeic acid, gallocatechin, and several flavonoid glycosides suggests that the mulberry extracts are rich in polyphenolic metabolites typically associated with plant defence and antioxidant activity. Among the quantified compounds, chlorogenic acid was the predominant component in the MNE (7.0487 mg/g dried extract), highlighting it as a major contributor to the phenolic profile. This compound is widely recognized for its antioxidant, anti-inflammatory, and potential metabolic regulatory effects. In contrast, the MAE showed higher levels of several other phenolics, particularly isochlorogenic acid, caffeic acid, and cyanidin-3-glucoside.

Anthocyanins such as cyanidin-3-glucoside and cyanidin-3-O-rutinoside were detected in both extracts, confirming the presence of pigmented flavonoids responsible for the characteristic coloration of mulberry fruits. These compounds are particularly important due to their documented antioxidant and cardioprotective effects [40]. Additionally, several flavonoid glycosides including hyperoside, rutoside, and luteolin-7-glucoside were identified, indicating that glycosylated flavonoids form a significant fraction of the mulberry phenolic matrix. These molecules are often associated with enhanced solubility and biological activity in plant extracts.

Notably, t-ferulic acid exhibited a considerably higher concentration in MNE extract (2.2771 mg/g dried extract) compared to MAE extract, suggesting that extraction conditions may influence the recovery of specific hydroxycinnamic derivatives. Meanwhile, flavonoid aglycones such as luteolin and kaempferol were detected at moderate levels, further supporting the diversity of flavonoid structures in the extract.

The chemical composition of extracts depends on the environmental conditions where these trees grow [6]. According to the general quantitative analysis, the MNE is richer in polyphenols than MAE. This data is similar or slightly different from those presented by other researchers because the solvents or methods used were different [41,42]. Talita et al. demonstrated that, by using ultrasound extraction, the chlorogenic acid content increased 2.5 times [43]. In another study, Fauzi et al. noticed that the *Morus alba* leaves extract obtained by microwave extraction using 60% ethanol contain more flavonoid glycosides, and some phenolic acids are separated in larger quantities [42].

If we compare our results with those presented by Li et al., we observe that the leaves contain more phenolic compounds than fruits, and the polyphenolic content depends on the mulberry varieties. From a quantitative point of view, the most important polyphenols identified in fruits are chlorogenic acid (0.246–2.518 mg/g dried fruits) and rutin (0.094–1.696 mg/g dried fruits) [44].

Oxidative stress results from an imbalance in oxidative processes caused by several factors, including increased endogenous oxidation and auto-oxidation processes, reduced concentrations of endogenous antioxidants, inactivation of antioxidant enzymes, decreased synthesis of antioxidant enzymes, and increased levels of pro-oxidants, such as transition metals [45].

Hydroxyl radicals are among the most reactive oxygen species, generated primarily through the Fenton reaction in the presence of Fe^2+^ ions. These radicals exhibit extremely high chemical reactivity and attack both intracellular and extracellular biomolecules [46]. Hydroxyl radicals are also produced by microglial cells and are involved in neuroinflammatory processes that contribute to neurodegeneration. Sometimes, cells lack enzymatic mechanisms capable to destroy hydroxyl radicals; therefore, antioxidant compounds are required to stabilise these species and reduce their harmful effects. At low concentrations, hydroxyl radicals also are involved in programmed cell death [47].

Uncontrolled oxidative processes induced by hydroxyl radicals lead to structural and functional alterations at both cellular and subcellular levels, triggering various pathological phenomena such as: inflammation, cardiovascular diseases, diabetes, neurodegenerative diseases and carcinogenesis [35,48].

MAE and MNE contain polyphenols, which, due to their hydroxyl groups, can donate protons and electrons and neutralize hydroxyl radicals. These polyphenols can also chelate Fe^2+^ ions involved in the synthesis of hydroxyl radicals through the Fenton reaction. A previous study using MAE and MNE showed that these extracts can chelate Fe^2+^ and reduce Fe^3+^ to Fe^2+^, and thus they reduce or block the pro-oxidant effects of these ions [39].

The chemical composition of extracts, especially the polyphenols content, is directly correlated with the observed antioxidant properties of extracts. Many studies have highlighted the correlation between polyphenol content and the antioxidant action of extracts, indicating that this activity is enhanced when polyphenols with a great number of hydroxyl groups are presented [49,50,51,52].

The oxidation of plasma lipids can be induced by transition metals, predominantly involving ferrous or cupric ions. The primary oxidation products possess high reactivity, further propagating oxidative chain reactions. The extracts that are able to block transition metals will decrease the rate of lipid oxidation [53].

The brain uses approximately 20% of the body’s oxygen and is therefore at increased risk of generating reactive oxygen species (ROS) and reactive nitrogen species (RNS). These compounds induce oxidation of polyunsaturated fatty acids, protein oxidation, DNA damage, mitochondrial impairment, and neuronal death [54].

Similar with plasma lipids, in the brain unsaturated lipids could be oxidated by metals. Peroxidation of unsaturated lipids generates products involved in the aging process or neurodegenerative disorders. Furthermore, the oxidative degradation of unsaturated fatty acids within the membrane phospholipid bilayer compromises structural integrity and selective permeability [55,56].

MNE, MAE and morin reduce plasma lipid oxidation and decrease the synthesis of compounds that react with thiobarbituric acid. Our previous study indicates the presence in extracts of polyphenols such as chlorogenic acid (highest quantity), gallic acid, quercetin, isoquercetin and morin [39]. All these compounds have 3–4 hydroxyl groups that could stabilise the ROS and block the peroxidation process. The flavonoids in the analysed extracts, such as quercetin and isoquercetin, which have an OH group in the C nucleus near the keto group, can also chelate copper ions, which have a pro-oxidant effect and trigger the lipid peroxidation process [50,57].

Antioxidants that inhibit lipid peroxidation reduce the formation of carbonyl derivatives, which can interact with proteins and alter their biological functions [45,58]. *Morus* sp. extracts also block lipoxygenase activity, an enzyme that is also involved in lipid peroxidation [39].

Oxidative stress induces mitochondrial dysfunction, leading to reduced ATP synthesis required for cellular processes, increased beta-amyloid accumulation, and enhanced formation of beta-amyloid aggregates. In addition, beta-amyloid itself may stimulate the generation of ROS within mitochondria, resulting in mitochondrial damage and neuronal death [15].

Neurodegenerative processes and especially Alzheimer’s disease are complex phenomena in which numerous mechanisms are involved, including oxidative ones. The antioxidant tests performed in this study, as well as those performed in previous studies, have highlighted the ability of the two extracts to act as antioxidants through several mechanisms. Indirectly, these extracts may also have anti-inflammatory effects, knowing that oxidative stress is correlated with neuroinflammation [59,60].

Acetylcholine and the normal functioning of cholinergic synapses are essential for neuronal communication and play a fundamental role in cognitive processes, including learning and memory. Cholinergic dysfunction in Alzheimer’s disease begins early in the course of the disease, worsens with disease progression, and results in impaired memory, reduced attention, and diminished learning capacity [18,61]. Beta-amyloid may reduce choline uptake into neurons, thereby decreasing both the synthesis and release of acetylcholine [61].

The ability of AChE to interact, through the hydrophobic groups from the peripheral anionic site, with amyloid beta and to promote the formation of amyloid fibrils and senile plaques also indicates its non-cholinergic involvement in the pathology of Alzheimer’s disease [62]. Blocking the anionic site will reduce the involvement of the enzyme in the development of senile plaques [63].

Phenolic compounds from MAE and MNE interact with amino acid residues from the active site of cholinesterases by hydrogen bonding, hydrophobic and π–π interaction. The number of interaction points depends on the number of hydroxyl groups, and this will influence the intensity of enzyme inhibition [36]. Inhibition of cholinesterases, particularly acetylcholinesterase, increases acetylcholine concentration in the synaptic cleft, thereby reducing cognitive deficits in patients with Alzheimer’s disease.

In the brains of healthy people, the ratio between acetylcholinesterase (AChE) and butyrylcholinesterase (BChE) is approximately 4:1, whereas in the brains of patients with Alzheimer’s disease, AChE levels decrease by nearly 50%, while BChE levels may double [38,64]. Analysing these data, together with our results, is very important for identifying extracts able to block both enzymes, with particularly emphasis on BChE, whose activity is elevated in patients with cognitive impairment. MNE inhibited AChE by 9.85% more than BChE, while MAE and morin showed greater inhibition of BChE by 5.03% and 10.44%, respectively.

The monomeric form of the acetylcholinesterase has been identified in the brains of transgenic mice that overproduce beta-amyloid similar to that found in the brains of Alzheimer’s disease patients. This monomeric form was observed to promote amyloid aggregation, leading to the formation and growth of insoluble fibrils and to the acceleration of neurotoxic processes [65,66].

Inhibition of cholinesterase represents a therapeutic approach in Alzheimer’s disease with acetylcholine level increasing, but at the same time a decrease of beta-amyloid deposition into the brain is observed [62]. Other studies have demonstrated the ability of certain plant compounds to inhibit butyrylcholinesterase more strongly than acetylcholinesterase [67,68]. For instance, moracin S isolated from *Morus radix* has the ability to block AChE (IC_50_ is over 30 µM) and BChE (IC_50_ is 7.22 ± 0.22 µM) [69].

Plant extracts that are rich in polyphenols induce dose-dependent inhibition of cholinesterase. The degree of inhibition depends on the number of hydroxyl groups present in the polyphenol structure. Glycosylated forms may also exhibit inhibitory activity due to their increased hydrophilicity and their enhanced ability to penetrate toward the active site of cholinesterase. The formation of hydrogen bonds with functional groups located within the anionic site of the enzyme blocks substrate access to the catalytic active centre [70]. Polyphenols identified in our extracts [39] and morin have three or four hydroxyl groups able to interact with functional groups from the active site of both enzymes.

The differential inhibitory activity of the tested extracts and morin toward the two cholinesterases may also be explained by structural differences within the anionic site of the enzymes. The amino acid composition of the anionic site is slightly different, thereby influencing both inhibitor interactions and substrate access to the catalytic site. Furthermore, the active-site gorge of butyrylcholinesterase is longer than that of acetylcholinesterase, resulting in different substrate accessibility to the active site [71,72].

Inhibition of acetylcholinesterase contributes to the reduction of neuroinflammation, as increased synthesis of pro-inflammatory cytokines and decreased levels of anti-inflammatory cytokines have been observed in brain regions where the activity of this enzyme is elevated [64].

Zebrafish (*Danio rerio*) is a vertebrate model organism widely used today in neuroscience research due to its highly conserved nervous system, which shares significant similarities with that of mammals. Scopolamine is used in cognitive-related tests because it acts on muscarinic acetylcholine receptors and induces Alzheimer’s-like memory deficit. Scopolamine impaired short-term memory and learning ability, leading animals to spent less time in the novel arm in the Y-maze test [20]. Behavioural changes induced by scopolamine and reversed by natural or synthetic compounds are very important in studies regarding the identification of new therapies for neurodegenerative diseases or cognitive impairments [22].

The Y-maze is used to assess short-term memory. The test was developed for mice or rats and is adapted for zebrafish by modifying the used device. The animals explore the arms of the device depending on the spatial working memory. An animal with intact working memory will remember the arms previously visited and has the curiosity to explore new spaces [23]. Spatial working memory is important for storage and use of spatial information in short-term memory and is related to cognitive decline. The patients with impairment of spatial working memory have mild cognitive impairment or Alzheimer’s disease. These patients have slower memory, lower accuracy, and have problems with processing the spatial information. The rate of losing spatial working memory is related with the worsening of Alzheimer’s disease [73,74]. In our study the extracts and morin demonstrated the ability to partially restore the spatial memory.

MAE and MNE treatment significantly attenuated the Sco-induced memory deficit. Both extracts, used in low doses and high doses (MAE-L, MAE-H, and MNE-L, MNE-H, respectively), markedly increased the time spent in the novel arm compared with the Sco group, with the high dose (MNE-H) exerting a more pronounced restorative effect, approaching the values observed in the control and galantamine-treated groups. Additionally, zebrafish treated with morin showed a significant increase in time spent in the novel arm compared with Sco-treated fish, further supporting the involvement of flavonoid-mediated procognitive mechanisms.

Regarding locomotor performance, Sco-treated zebrafish exhibited no significant changes in total distance travelled, while turn angle increased significantly compared to the control group, reflecting altered exploratory dynamics. Administration of MAE, MNE, and morin significantly improved locomotor parameters in Sco-exposed zebrafish. Both extracts (MAE, MNE) significantly increased turn angle values. The high dose (MAE-H; MNE-H) produced a more pronounced effect, indicating a dose-dependent behavioural improvement.

Both extracts and morin mitigate scopolamine-induced cognitive impairment and behavioural alterations in the Y-maze task, with the higher dose displaying superior efficacy.

Many chemical compounds and plant extracts have been used to evaluate spatial working memory in order to identify those that could be used to treat cognitive impairment and Alzheimer’s disease. Different plant extracts or volatile oils are analysed, and the results depend on the chemical composition of these and the doses used. Choi et al. noted that *Allium hookeri* hydroethanolic extract improves visual working memory by different mechanisms: it increases the activity of the cholinergic system, reduces pro-inflammatory cytokine synthesis, and reduces beta-amyloid synthesis [21]. In another study, the animals were treated with *Salvia* sp. Extracts, and they spent significantly more time in the novel arm compared to the familiar arm in the Y-maze test [75].

The novel tank diving test (NTT) is used to study anxiety-like behaviour in zebrafish. In this test the anxiety is directly correlated with the time spent by animals at the bottom of the tank. The animals exposed to compounds able to decrease anxiety have the tendency to spent more time in the top part of the tank [76].

Scopolamine induced anxiogenic effects by increasing the latency period required for fish to begin vertical exploration of the tank, reducing the time spent by fish in the top of the tank, and by decreasing the distance travelled by fish in the top of the tank.

Treatment with *Morus alba* extract (MAE-L and MAE-H), *Morus nigra* extract (MNE-L and MNE-H) and morin significantly attenuated the anxiogenic effects induced by scopolamine. Both doses increased the time spent in the top zone compared with Sco-treated fish and significantly reduced freezing duration. The high doses (MAE-H, MNE-H) exhibited a stronger restorative effect across parameters, suggesting a dose-dependent anxiolytic-like response.

Imipramine (IMP, 71 μM), used as a positive reference drug, significantly increased time spent in the top zone and reduced freezing duration compared with Sco-treated fish, confirming its anxiolytic-like activity. The behavioural improvements observed following MAE, MNE and morin administration were comparable to those induced by imipramine. Collectively, these findings indicate that MAE, MNE and morin exert anxiolytic-like effects in Sco-treated zebrafish, accompanied by normalization of locomotor activity for MAE extract, and without markedly affecting basal locomotor performance for MNE extract.

The NOR test is used to evaluate the preference of zebrafish for novelty, and its results are correlated with reference memory. Curiosity for new objects, new places or new events from general life is important for the evolution of the patients with Alzheimer’s disease [77].

The administration of extracts (MAE, MNE) significantly attenuated memory deficit in the NOR test. Both extracts, in low (MAE-L, MNE-L) and high doses (MAE-H, MNE-H), respectively, increased preference percentages compared to the Sco-treated group, with the high dose producing a more pronounced restorative effect.

The improvement observed in the MAE-H and MNE-H groups approached galantamine values, indicating recovery of recognition memory performance. Galantamine (GAL, 3.5 µM), used as a positive reference drug, significantly increased preference for the novel object compared to Sco-treated zebrafish, confirming its procognitive activity.

These findings are consistent with the behavioural improvements observed in the Y-maze test. Overall, the results demonstrate that *Morus alba* and *Morus nigra* extracts mitigate scopolamine-induced recognition memory impairment, with evidence of dose-dependent efficacy.

These behavioural changes suggest an improvement in spatial memory as a result of the action of the two extracts and morin at hippocampus and prefrontal cortex level. Similar effects have been observed by other researchers using different plant extract such as coriander oil [20], *Lantana camara* extract [24], fermented bran rice extract [25], and *Yucca schidigera* bark extract [29]. In all these studies, the observed effects are correlated with the dose used and the chemical composition of the plant extracts or volatile oils.

In several studies investigating the effects of *Morus* sp. extracts on cognitive decline or the progression of Alzheimer’s disease, the fruits have been predominantly used, as they are widely consumed in human nutrition. These researches indicate the capacity of fruit extracts to block the acetylcholinesterase activity or to reduce the neuroinflammation [78]. In another study, polyphenol-rich extracts from *Morus nigra* fruits reduced beta-amyloid synthesis and Alzheimer’s disease symptomatology by modifying the intestinal microbiome and the gut–brain axis [79].

According to our previous published data, the tested extracts also exhibit inhibitory activity against enzymes involved in carbohydrate digestion, such as alpha-amylase and alpha-glucosidase [39]. These effects represent an additional benefit, considering that current studies support a direct or indirect causal relationship between diabetes mellitus and Alzheimer’s disease [80,81].

Alzheimer’s disease therapy sometimes produces only minor symptomatic improvement and is often accompanied by adverse effects that are not well tolerated by patients. Cholinesterase inhibitors produce adverse effects through indirect stimulation of the parasympathetic nervous system, including nausea, vomiting, diarrhoea, and bradycardia [82]. These effects reduce patient adherence to therapy and increase the risk of disease progression. Therefore, plant extracts with similar or improved therapeutic effects but with minimal adverse reactions represent a promising therapeutic option [83,84,85,86].

By correlating these positive effects with the results of antioxidant and cholinesterase inhibition tests, we can consider that the neuroprotective effect may be determined by antioxidant protection, but also by increasing the amount of acetylcholine in the cerebral synapses. The results of our study indicate the neuroprotective effects of *Morus alba* and *Morus nigra* extracts.

## 5. Conclusions

Our study highlighted the antioxidant and enzyme inhibitory activities of *Morus* extracts and morin. Concurrently, their capacity to attenuate cognitive impairments induced by scopolamine administration was observed. The antioxidant and cholinesterase inhibition tests were performed in vitro, and even if we cannot state that these mechanisms are involved in neuroprotection, the results will be used for the future development of an in vivo experimental protocol in which the antioxidant and enzyme inhibitory effects of the extracts will be analysed.

The efficacy of therapy with acetylcholinesterase inhibitors depends on the number of functional synapses in the brain of patients with Alzheimer’s disease. As the disease progresses and the number of functional synapses decreases, and the efficacy of these inhibitors declines. Therefore, the identification of compounds capable of inhibiting cholinesterases and their administration as early as possible in patients with cognitive impairment represent an important strategy for improving the quality of life of patients with cognitive deficits or Alzheimer’s disease.

Plant extracts have the advantage of combining anticholinesterase and antioxidant activities, as oxidative stress plays a key role in the onset and worsening of Alzheimer’s disease. Antioxidant compounds may delay the onset and slow the progression rate of Alzheimer’s disease.

Moreover, the enzyme inhibition properties of plant-derived compounds may be useful for the development of semisynthetic derivatives with stronger inhibitory activity and improved ability to cross the blood–brain barrier. In addition, currently used drugs may be combined with vegetal extracts in order to reduce adverse effects.

## Figures and Tables

**Table 1:** The most important compounds quantified in the investigated extracts.

Compound	Concentration (mg/g Dried Extract)	
	MNE	MAE
Gallocatechin	0.4412	0.7491
Caffeic acid	1.5303	2.2285
Cyanidin-3-glucoside	1.3970	2.2406
Chlorogenic acid	7.0487	3.2612
Quercetin-3-beta-D-glucoside	0.3006	0.1217
Rutoside	0.6837	0.3808
Hyperoside	2.7243	2.6986
Luteolin-7-glucoside	1.0652	1.7353
Isochlorogenic acid	0.3974	2.5429
Luteolin	1.0322	1.2806
Kaempferol	0.5064	-
Cinnamic acid	0.1109	1.1076
Cyanidin-3-O-rutinoside	0.2057	0.2631
t-ferulic acid	2.2771	0.0417

MAE—*Morus alba* extract, MNE—*Morus nigra* extract.

**Table 2:** The IC_50_ values for antioxidant tests and cholinesterase inhibition tests.

Sample	IC_50_ (µg/mL)			
	Hydroxyl Radical Scavenging Assay	Lipid Peroxidation Inhibition Assay	Acetylcholinesterase Inhibition Assay	Butyrylcholinesterase Inhibition Assay
MNE	87.99 ± 2.93 ^a^	25.31 ± 2.54	24.34 ± 0.86	27.00 ± 2.84
MAE	100.67 ± 2.77 ^b^	29.85 ± 0.97 ^a^	46.87 ± 2.16 ^e^	44.51 ± 2.54 ^e^
Morin	111.54 ± 2.77 ^c^	34.56 ± 1.47 ^b^	26.84 ± 0.99 ^d^	29.97 ± 2.78 ^d^
Vitamin C	74.20 ± 1.75	24.10 ± 2.10	-	-
Galantamine	-	-	21.50 ± 0.85	22.25 ± 1.27

MAE—*Morus alba* extract, MNE—*Morus nigra* extract; The results are presented as mean ± standard deviation. ^a^ *p* < 0.01 sample vs. vitamin C; ^b^ *p* < 0.001 sample vs. vitamin C; ^c^ *p* < 0.0001 sample vs. vitamin C; ^d^ *p* < 0.01 sample vs. galantamine; ^e^ *p* < 0.0001 sample vs. galantamine.

## Data Availability

The original contributions presented in this study are included in the article. Further inquiries can be directed to the corresponding authors.

## Abbreviations

DMSO	dimethyl sulfoxide
DTNB	5,5′-bis-2-nitrobenzoic acid
FDA	Food and Drug Administration
MAE	*Morus alba* extract
MNE	*Morus nigra* extract
NMDA	N-methyl-D- aspartate
NOR	Novel object recognition test
NTT	Novel tank diving test
ROS	Reactive oxygen species
RPM	Revolutions per minute
MAE	*Morus alba* extract
MNE	*Morus nigra* extract
RP-UHPLC-PDA	Reversed-phase high-performance liquid chromatography with photodiode array detection
